# Cervicothoracic trans-spinal magnetic stimulation: effect of coil position and orientation on spinal motor evoked potentials

**DOI:** 10.1007/s00221-026-07390-y

**Published:** 2026-08-31

**Authors:** Lei Zhu, Sophia G. Nopoulos, Kesten M. Anderson, Richard K. Shields, Stacey L. DeJong

**Affiliations:** Department of Physical Therapy & Rehabilitation Science, Roy J. and Lucille A. Carver College of Medicine, University of Iowa, 3500 Health Sciences Academic Building, Iowa City, IA 52242-1308 USA

**Keywords:** Spinal stimulation, Magnetic stimulation, Induced current direction, Motor evoked potentials

## Abstract

Stroke is a leading cause of long-term adult disability, and over 80% of survivors have persistent upper limb motor impairment. Spinal electrical stimulation has shown therapeutic potential after neurological disorders. Post-stroke applications have largely relied on invasive implants that limit clinical adoption, and non-invasive high intensity electrical stimulation may be intolerable for those with residual sensory function. Trans-spinal magnetic stimulation offers a non-invasive alternative capable of modulating residual sensorimotor pathways. Fundamental parameters, such as coil position and orientation, remain under-investigated. Two experiments were conducted to fill this gap. Experiment 1: 19 healthy adults underwent spinal mapping involving 30 cervicothoracic sites (3×10 grid). Responses were recorded from 6 upper limb muscles bilaterally at suprathreshold intensity using a rostral-left induced current direction. Friedman ANOVAs revealed significant medial–lateral and rostral–caudal variation in amplitude of spinal motor evoked potentials (sMEPs): ipsilateral>midline>contralateral, with arm responses peaking rostrally, wrist at mid-rows, and hand caudally. Experiment 2: 20 healthy adults received stimulation at three horizontal cervicothoracic sites using eight current directions spaced 45° apart. Friedman ANOVAs indicated a bilateral mirror-symmetrical pattern. The largest responses from left-sided muscles were elicited with a rostral-left current direction achieved by rotating the coil handle 45° counterclockwise from the caudal direction. Likewise, the largest right-sided muscle responses were elicited with a rostral-right current direction achieved by rotating the coil handle 315° counterclockwise. Coil position and orientation are critical determinants of sMEP amplitude. These results provide a foundational framework to further investigate cervicothoracic magnetic stimulation as a potential non-invasive neuromodulatory intervention.

## Introduction

Spinal stimulation has been investigated as a promising neuromodulatory method for upper limb rehabilitation, with evidence suggesting its therapeutic potential as a priming intervention before motor training. Delivered invasively or non-invasively, spinal electrical stimulation is proposed to engage neuronal circuits and enhance spinal excitability, contributing to functional recovery for people with neurological disorders, including those with spinal cord injury (SCI) (Angeli et al. 2018; Inanici et al. 2021) and stroke (Powell et al. 2023). These encouraging results support the potential therapeutic value of trans-spinal magnetic stimulation (TSMS) as a novel non-invasive alternative, however identifying optimal parameters in healthy individuals is an essential step before translating this approach to clinical populations.

TSMS is an approach adapted from the well-established technique of transcranial magnetic stimulation (TMS), first introduced by Barker et al. (Barker et al. 1985), but applied specifically over the spine. Applying repetitive protocols with frequencies ranged from 5 to 20 Hz, TMS targeting the primary motor cortex (M1) has shown promising effects in enhancing neuroplasticity and facilitating motor recovery (Dionisio et al. 2021; Hensel et al. 2019; Huang et al. 2005; Wang et al. 2020). Nevertheless, recent studies have raised concerns about the stability and reliability of these effects (Magnuson et al. 2023). One possible explanation is that efficacy may be constrained by stimulation of compromised corticospinal pathways. As an alternative approach, TSMS may potentially address this limitation by directly or indirectly targeting spinal pathways involved in sensorimotor processing through neural substrates other than the descending pathways (e.g., ascending tracts, intraspinal networks, and monosynaptic reflexes) (Freyvert et al. 2018; Inanici et al. 2021; Powell et al. 2018, 2023). Building on this rationale, preclinical work in animal models has provided preliminary evidence for the feasibility of TSMS in improving motor control (Chalfouh et al. 2020). Using specially designed magnetic coils, repetitive TSMS at 10 Hz for 14 days in a mouse model of SCI significantly improved hindlimb movement quality and speed compared with controls. Functional gains were evident as marked reductions in hindlimb errors and overall crossing time, supporting potential investigation of TSMS in clinical applications.

To guide future TSMS research, it is essential to first investigate the stimulation parameters, particularly where and how to place the coil. Insights from TMS studies targeting M1 could therefore provide useful references. For instance, cortical mapping has shown that activation of specific muscles can be achieved by moving the coil to the corresponding cortical region (Proelss et al. 2025; Tardelli et al. 2022; Yuasa et al. 2022), suggesting the importance of stimulation location as a parameter. Moreover, the effects of coil orientation have been examined across multiple muscles groups (Bashir et al. 2013; Ma et al. 2023; Smith et al. 2017; Souza et al. 2018), with consistent evidence that largest responses were elicited when the induced current flow was oriented perpendicular to the targeted neurons. In M1, this corresponds to a posterior-anterior induced current oriented perpendicular to the postcentral gyrus (Richter et al. 2013), with the coil handle held at a 45 ° angle to the mid-sagittal line. This is now widely adopted as the standard orientation for TMS targeting, recognizing the importance of stimulation orientation as a parameter. However, due to anatomical differences, these findings cannot be directly transferred to TSMS, indicating the necessity of establishing these parameters in TSMS-specific studies.

In contrast to the extensive work on stimulation parameters in TMS, existing investigations at the spinal level remain limited. Early work demonstrated that responses to cervical magnetic stimulation vary with stimulator placement, highlighting the importance of stimulation configuration (Schmid et al. 1990). However, systematic characterization of coil position and orientation across the cervicothoracic region and multiple bilateral muscles remains limited. The cervicothoracic region has been specifically targeted as it provides access to upper limb motor pathways, yet it has not been systematically mapped; instead, most spinal stimulation studies have relied on generalized myotome charts and palpation of landmarks such as the spinous process of the seventh cervical vertebra (C7) (Knikou 2013a, 2017). In addition, early investigations of the effects of coil orientation on amplitude of TSMS-induced spinal motor evoked potentials (sMEPs) provide preliminary insights but have been restricted to broad directional comparisons, for example, transverse vs. longitudinal (coil handle aligned perpendicular vs. parallel to the spinal axis) (Mills et al. 1992); or counterclockwise vs. clockwise current within the coil (Ugawa et al. 1989). Later studies examined specific coil angles but remained confined to different spinal segmental levels. Considering the longitudinal alignment of spinal nerves after exiting the neural foramina, it is particularly important to evaluate coil placement along medial–lateral distances. Taken together, these limitations highlight the need for systematic spinal mapping and for assessing coil orientation across a wider range of angular orientations and medial–lateral locations.

The objective of this study was to fill critical knowledge gaps regarding stimulation parameters of TSMS at the cervicothoracic spine. Specifically, we investigated how coil position and orientation influence motor responses in the arm, wrist, and hand, as quantified by the amplitudes of sMEPs. We hypothesized that coil position and orientation would significantly modulate sMEPs, and that optimal parameters could be identified to elicit the maximal responses across targeted muscle groups.

## Methods

### Participants

The study included two separate experimental visits. Experiment 1: the spinal mapping visit, to address the effects of stimulation location; and Experiment 2: coil orientation visit, to subsequently investigate the effects of induced current direction on sMEPs. These are hereafter referred to as the “spinal mapping visit” and the “rotation visit”. Each participant completed the spinal mapping visit first, followed by the rotation visit, with at least 24 h between visits (mean ± SD: 9.33 ± 10.23 days).

Nineteen healthy adults aged ≥ 21 years (10 females; age: 38.7 + 18.6 years; 18 right-handed, 1 with mixed handedness) participated the spinal mapping visit (experiment 1). The same cohort participated in the subsequent rotation visit with one withdrawal (male, 50 years old, right handed). Two additional right-handed healthy male subjects were enrolled to achieve a total sample of 20 participants for the rotation visit (experiment 2) (10 females; age: 33.01 ± 25.56 years; 19 right-handed, 1 with mixed handedness).

For both visits, participants were recruited if they had no previous injuries or medical history related to neurologic or musculoskeletal disorders and had no contraindications to TMS (Rossini et al. 2015). Upper limb function was screened to exclude those outside the normal range for healthy individuals. Assessments included active range of motion of bilateral shoulders, elbows, wrists, and fingers, a thumb–index finger dexterity task, sensory testing of the index finger with Semmes–Weinstein monofilaments, and grip and pinch strength measurements using Jamar dynamometers.

Written informed consent was obtained prior to participation, in accordance with a study protocol approved by the University of Iowa Institutional Review Board (202109535).

### Instrumentation

#### Delsys EMG system

Electromyographic (EMG) signals were collected from six upper limb muscles bilaterally using wireless surface Trigno EMG sensors (Delsys Trigno, Natick, MA, USA) with bandwidth 20–450 Hz frequency. Following skin preparation with alcohol wipes, standard Trigno electrodes were placed over the muscle bellies of the flexor carpi radialis (FCR), extensor carpi radialis longus (ECR), biceps brachii (BB), triceps brachii (TB), and abductor digiti minimi (ADM) bilaterally. Mini Trigno sensors were applied over the first dorsal interosseous (FDI) bilaterally.

#### Magnetic stimulation

Single-pulse magnetic stimulation was delivered using a MagVenture stimulator (MagVenture MagPro X100 with MagOption; Alpharetta, GA, USA) and a figure-of-eight focal coil (C-B60, MagVenture, Alpharetta, GA, USA). The stimulator was set to a “Biphasic” waveform and 'Power' mode to achieve greater focality and higher stimulation power. Specifically, the biphasic waveform was selected because prior study indicated the biphasic waveform can yield lower motor threshold compared to the monophasic and half-sine waveforms (Sommer et al. 2006). “Reverse current” mode was applied to align the direction of the induced current opposite the direction of the coil handle.

#### Spinal neuronavigation system

**Fig. 1:**
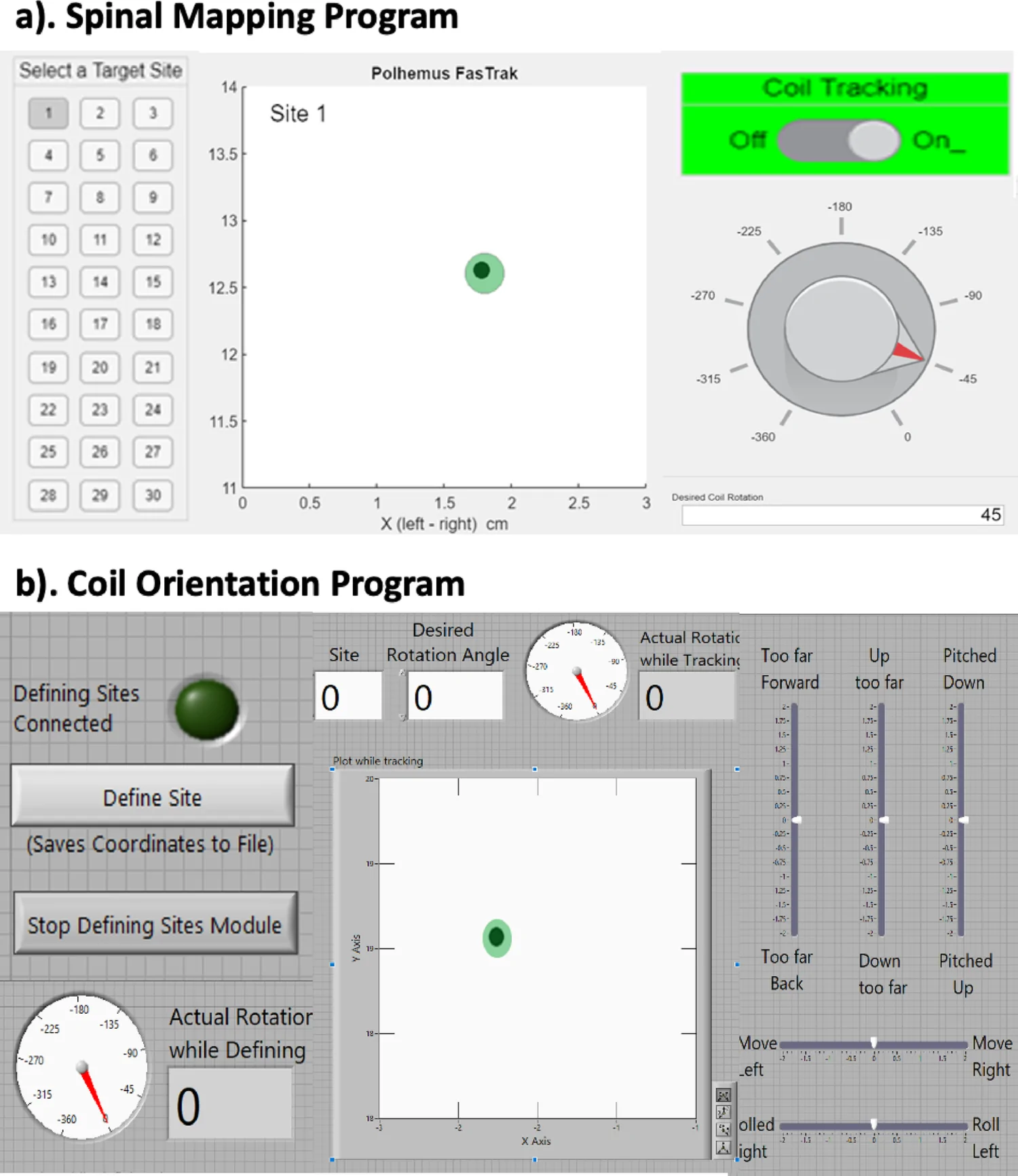
Spinal Neuronavigation System This figure illustrates a custom-developed spinal neuronavigation system built in LabVIEW and used throughout stimulation. Two programs were designed for spinal mapping and coil orientation, respectively. **a** Spinal Mapping Program: Once the coil was visually aligned over the target location, any of the 30 stimulation sites shown in the left panel could be selected by clicking its corresponding number. The site’s coordinates then appeared in the central x–y graph, and the system tracked the real-time coil position and orientation during stimulation. **b** Coil Orientation Program: At the start of each session, stimulation sites were defined by aligning the TMS coil over the target while monitoring the real-time rotation angle display (left panel, “Actual Rotation while Defining” box). Once the displayed angle matched the intended orientation (e.g., 45°), the “Define Site” button was clicked. The system recorded the site number and corresponding spatial (x, y, z) and rotational (yaw, pitch, roll) coordinates, which were plotted as a black dot on the middle graph. Adjustable sliders in the right panel allowed fine-tuning of the coil position to ensure precise and consistent targeting during stimulation. In both programs, a green dot appeared when the coil was within 2 mm of the target site and within 2° of the intended orientation

Coil placement during stimulation, including the coil location and orientation, was tracked continuously using a custom-developed neuronavigation system using the FASTRAK 6D electromagnetic motion tracking system (FASTRAK, Polhemus, Colchester, Vermont, USA) combined with custom-written software (LabVIEW, National Instruments, Austin, TX, USA). Separate custom LabVIEW programs were developed to support the spinal mapping and rotation experiments (Fig. 1).

The FASTRAK system tracked two reference sensors, each providing real-time position coordinates (x, y, z) and orientation angles (yaw, pitch, roll). One reference sensor was mounted on the coil, offset from the coil center by 15 cm, and was maintained in that position consistently across all participants and all visits. Another reference sensor was taped to the participant’s skin, in the midline over the spinous process of the fifth thoracic vertebra. This location was chosen because it was caudal enough to allow for alignment of the MagVenture coil more rostrally over the cervicothoracic spine, and because it was minimally affected by the movement associated with breathing. During stimulation, accurate and consistent alignment over each stimulation site was achieved by tracking the two reference sensors and replicating the relative distances and rotation angles between them in real-time. Stimulation was delivered only when the coil was aligned within 0.5 mm of the target location and within 2° of the desired rotation angle.

### Experimental protocol

#### Experiment 1: spinal mapping protocol

**Fig. 2:**
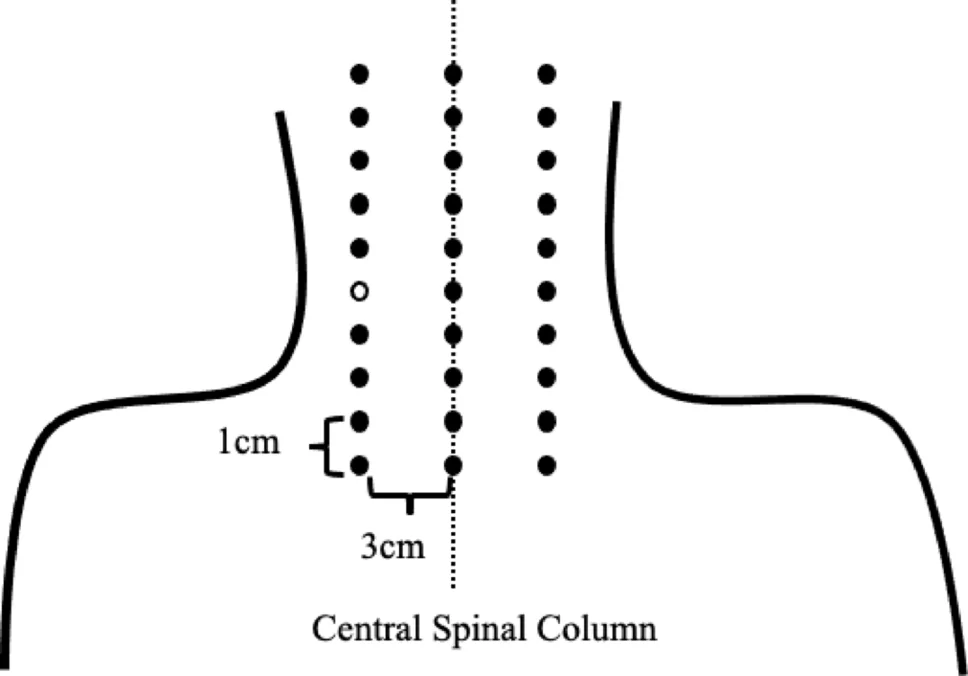
Mapping of 30 Stimulation Sites at the Cervicothoracic Spinal Region The graph illustrates the 3×10 stimulation grid used during mapping testing, with each site separated by 1 cm within columns and columns spaced 3 cm apart. The middle column lies over the spinal midline, and the 6th row of the left column was designated as the hotspot for left wrist extensor, as indicated by the open circle

After EMG electrodes were applied as described above, the participant was seated in a comfortable chair in a semi-prone position with their head resting on a massage pillow (TravelMate Massage Kit, EarthLife, Vista, CA) and their arms relaxed on a table in front, with padding under the elbows, forearms and hands. The table height and pillow position were carefully adjusted to achieve full cervical flexion, which was necessary for optimal coil positioning. Experimental testing began with hotspot identification for the left ECR, chosen because its spinal innervation lies approximately midway rostrocaudally among all of the targeted arm and hand muscles (Greiner et al. 2021). The spinous processes were palpated to identify the midline, and a reference line was drawn 3 cm lateral to the midline on the left side using eyeliner. This distance was selected to ensure a clear spatial distinction between the spinal canal and dorsal root regions, based on the approximate 1.27 cm width of the cervicothoracic spinal cord (Bican et al. 2013). The left ECR hotspot was identified by moving the coil rostrally to caudally along the left lateral line in 1 cm increments initially, followed by progressively smaller increments until the site eliciting the largest sMEP for left ECR was identified. A 3 ×10 grid of 30 stimulation sites was marked on the skin with eyeliner, as illustrated in Fig. 2, with the left ECR hotspot designated as the sixth row in the leftmost column. Stimulation sites were sequentially labeled from 1 to 30, starting at the upper end of the left column. Using the conventional method, resting motor threshold (rMT) was determined at the hotspot site as the lowest stimulation intensity that evoked left ECR sMEP amplitudes greater than 50 µV in at least 5 out of 10 consecutive trials (Rossini et al. 2015).

Next, the grid site that produced the largest response in the left BB was located by stimulating each site in the left column at a near-threshold intensity. The left BB rMT was determined at that site and was used to determine the stimulus intensity used for spinal mapping. Thus although the location of the mapping grid was determined using the left ECR hotspot, stimulation intensity during mapping was calculated based on the left BB rMT. BB appeared to have a higher threshold than the other tested muscles, and therefore allowed a more complete assessment of the distribution of sMEPs across hand, forearm, and arm muscles.

Ten consecutive sMEPs were recorded from 12 upper limb muscles at each of the 30 stimulation sites in random order. Stimulation intensity was set at 120% of the left BB rMT. The figure-of-eight magnetic coil was held with the handle oriented caudally and 45° counterclockwise, creating a rostral-left induced current direction. Ten single pulses were delivered with inter-stimulus intervals of at least 6 s. The previously described spinal neuronavigation system was utilized throughout the stimulation to ensure accurate and consistent targeting.

#### Experiment 2: coil orientation protocol

**Fig. 3:**
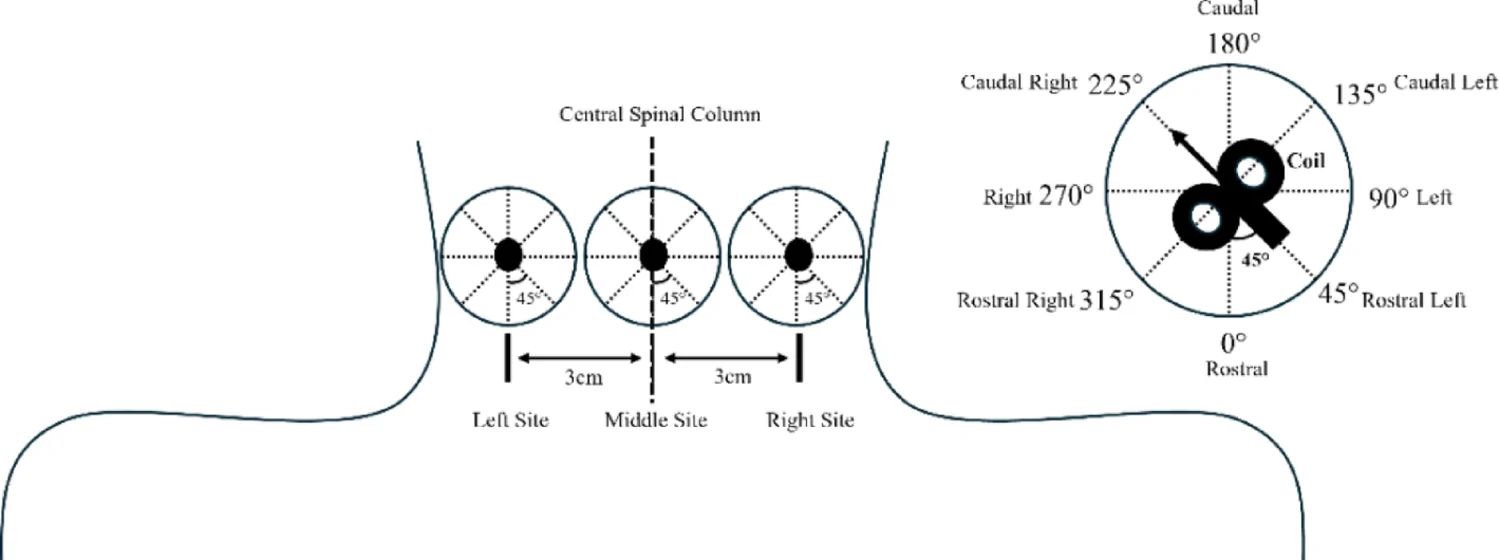
Example Graph for Coil Rotation Testing The graph illustrates the three tested stimulation sites (filled black dots) located at the cervicothoracic spine: the left site (hotspot for the left ECR), the middle site (aligned over the spinous process), and the right site (3 cm lateral to the middle). These three sites were located at the same rostrocaudal level. Each site was framed by an outline with four reference lines at 45° intervals to facilitate coil handle alignment in conjunction with the spinal neuronavigation system. Eight induced current directions were tested by rotating the coil handle along the guides, as shown in the right panel. The example demonstrates that when the coil handle was rotated to a 45° angle, delivery of a magnetic pulse induced a rostral–left current direction, as shown by the black arrow

The rotation visit involved testing eight induced current directions at three stimulation sites (Fig. 3). Upon arrival, participants underwent the same sequence of preparation procedures as in the spinal mapping visit: skin preparation, Delsys EMG placement, and positioning comfortably in the chair in a semi-prone posture.

The examination began by identifying the left ECR hotspot using the same approach as in the spinal mapping visit, systematically moving the coil rostrally to caudally along a line 3 cm lateral to the midline on the left. This left ECR hotspot was designated as the “left site” for testing. Two additional stimulation sites were identified at the same rostrocaudal level: the midline (“middle site”), and a site 3 cm lateral to the midline on the right (“right site”) (Fig. 3). These three sites were colinear in the frontal plane. The left and right sites were positioned symmetrically, presumably overlying posterior peripheral nerve roots.

**Fig. 4:**
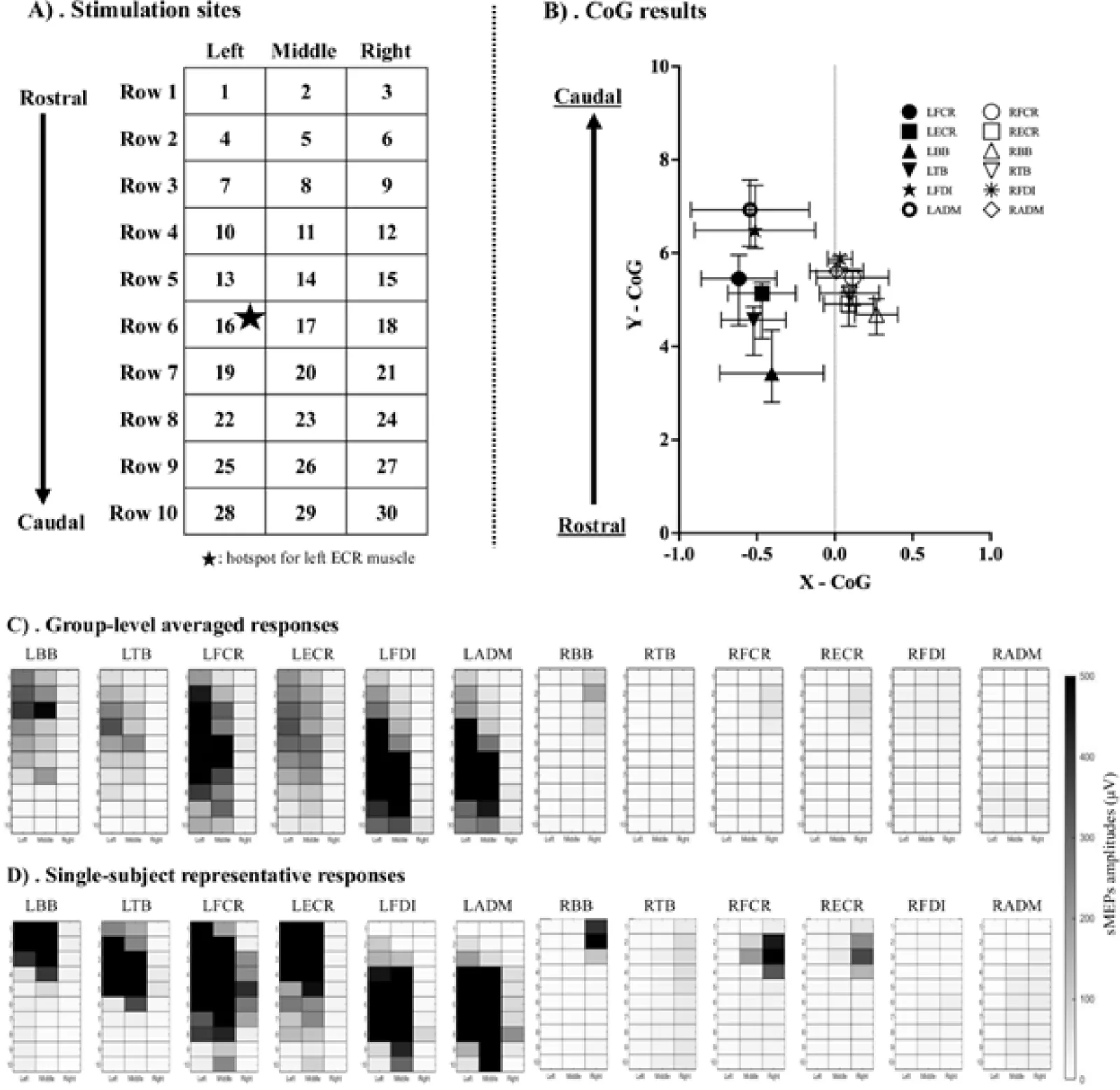
Spinal mapping results **A** Example layout of the 30 stimulation sites, labeled from Site 1 to Site 30 across 3 columns and 10 rows. Site 16 was identified as the hotspot for the left ECR muscle, located in the 6th row of the left column. **B** Group-level center of gravity (CoG) results across 12 bilateral upper limb muscles and 19 participants. Each point represents the group-level CoG location for each muscle on the stimulation grid. The x-coordinate indicates the mediolateral position of the CoG, and the y-coordinate indicates the rostrocaudal position of the CoG. Horizontal and vertical error bars represent the interquartile range (IQR) across participants for x-CoG and y-CoG, respectively. **C** Group-level heatmap showing sMEP amplitudes from each site, averaged across 12 muscles and 19 participants. Darker colors indicate larger responses. **D** Single-subject heatmap illustrating individual-level sMEP amplitudes across all sites and muscles. The observed medial–lateral and rostral–caudal patterns are consistent with the group-level results. Abbreviations: L-left (i.e., LFCR-left FCR), R-right

After the three sites were marked, the left ECR spinal rMT was determined at the left site, defined as the lowest intensity that elicited left ECR sMEP amplitudes > 50 µV in at least 5 out of 10 consecutive single pulses. To test the effect of induced current direction on sMEP amplitudes, eight coil handle rotation angles were selected, spaced 45° apart. The induced current directions included rostral (coil handle pointing caudally, 0°), rostral left (45°), left (90°), caudal left (135°), caudal (180°), caudal right (225°), right (270°), and rostral right (315°).

Ten consecutive magnetic pulses were delivered with the figure-of-eight coil held at each of the eight rotation angles and at each of the stimulation sites. Inter-pulse intervals were at least 6 s, to allow for adequate recovery of the stimulated neural circuit. The order of stimulation sites and rotation angles was randomized separately for each participant. Intensity of the stimulation was maintained at 120% of the spinal rMT of the left ECR muscle, as described above. Throughout the experiment, the locations of the three testing sites and the orientation of the magnetic coil were carefully tracked using the previously described spinal neuronavigation system, to ensure accurate and precise targeting.

### Statistical analysis

TSMS-induced sMEPs were analyzed using custom-written MATLAB software (The MathWorks, Inc., Natick, MA) to determine the peak-to-peak EMG amplitude during the interval from 5 to 50 ms following each stimulus. For each stimulation site tested during each visit, sMEPs from each muscle were averaged across 10 consecutive repetitions.

Because many of the muscles, sites, and orientations showed little to no response (sMEP amplitudes <50 µV), additional calculation was performed. For the spinal mapping visit (Experiment 1) specifically, center of gravity (CoG) coordinates were calculated across the cervicothoracic stimulation grid for each tested muscle. The x-CoG coordinate represented the mediolateral position across the three stimulation columns, with values ranging from − 1 to 1. The y-CoG coordinate represented the rostrocaudal position across the ten stimulation rows, with values ranging from 1 to 10. For each participant and muscle, x-CoG and y-CoG were calculated using the following weighted-average equations:

$$x-CoG = \frac{\sum (x-coordinate of site \times sMEP amplitude at site)}{\sum (sMEP amplitudes across all sites)}$$

$$y-CoG = \frac{\sum (y-coordinate of site \times sMEP amplitude at site)}{\sum (sMEP amplitudes across all sites)}$$

In the coil rotation visit (Experiment 2), peak-direction frequency was summarized for each muscle as the proportion of participants whose maximal sMEP response occurred at the same coil orientation.

Statistical analyses were performed using SPSS. Shapiro–Wilk tests indicated that for both visits, sMEPs and CoG data were not normally distributed (*p* < 0.05). A non-parametric Friedman ANOVA was performed for sMEP amplitudes to analyze differences in TSMS-induced responses across stimulation sites, muscles, and coil rotation angles. Wilcoxon signed-rank tests with Bonferroni correction were conducted for post-hoc pairwise comparisons where indicated. In experiment 1, significance thresholds were set at 1) *p* < 0.0017 (0.05/30) for comparisons across 30 stimulation sites; 2) *p* < 0.017 (0.05/3) for comparisons across 3 medial to lateral columns, and 3) *p* < 0.005(0.05/10) for comparisons across 10 rostral to caudal rows. In experiment 2, significance threshold of *p* < 0.00139 (0.05/36) was set for each Friedman test, and at *p* < 0.0018 (0.05/28) for the pairwise comparisons. For each of the 12 muscles, *p* < 0.004 (0.05/12) was set for the Friedman test and *p* < 0.017 (0.05/3) for the pairwise comparisons.

CoG coordinates were analyzed separately for the mediolateral and rostrocaudal directions. Statistical differences in CoG location across muscles were assessed using separate non-parametric Friedman ANOVA tests for x-CoG and y-CoG values. Post hoc pairwise comparisons were performed using Dunn’s multiple comparisons where indicated, with a Bonferroni adjusted significance level at *p* < 0.004 (0.05/11). Analyses were conducted using complete cases only. The right FCR muscle was excluded from the Friedman analysis because right FCR recordings from 5 of 19 participants were contaminated by background noise, resulting in incomplete repeated-measures data for this muscle.

## Results

TSMS was well tolerated by all participants, and no stimulation related discomfort or adverse event was reported. Descriptive results for both experiments are presented in Table 1.

**Table 1:** Descriptive results of spinal mapping and coil-orientation analyses

Muscle	Spinal mapping results (Experiment 1)					Coil orientation results (Experiment 2)			
	x-CoG (Median ±IQR)	y- CoG (Median ±IQR)	Peak site	Peak coordinates	Peak sMEP amplitudes (μV) (Median ±IQR)	Peak Orientation	Peak site	Peak sMEP amplitudes (μV) (Median ±IQR)	Peak orientation response %
RFCR	0.11 ± 0.23	5.28 ± 0.76	9	(1,3)	18.92 ± 25.13*	Rostral R	R	324.73 ± 616.14	73.33 (11/15)
RECR	0.09 ± 0.16	5.21 ± 0.74	3	(1,1)	16.51 ± 23.88*	Rostral R	R	160.50 ± 185.81	70 (14/20)
RBB	0.27 ± 0.14	4.98 ± 0.77	6	(1,2)	33.28 ± 77.12*	R	R	20.63 ± 12.66*	25 (5/20)
RTB	0.09 ± 0.19	5.44 ± 0.52	6	(1,2)	13.95 ± 27.1*	Rostral R	R	36.83 ± 81.39*	50 (10/20)
RFDI	0.03 ± 0.08	5.57 ± 0.26	21	(1,7)	14.55 ± 6.36*	R	R	330.68 ± 748.18	25 (5/20)
RADM	0.01 ± 0.17	5.51 ± 0.33	21	(1,7)	10.05 ± 7.53*	Rostral R	R	388.42 ± 1271.27	50 (10/20)
LFCR	−0.62 ± 0.24	5.45 ± 1.51	19	(− 1,7)	395.38 ± 501.31	Rostral L	L	985.01 ± 1081.90	95 (19/20)
LECR	−0.47 ± 0.22	5.04 ± 1.20	7	(− 1,3)	241.57 ± 221.13	Rostral L	L	241.06 ± 178.79	100 (20/20)
LBB	−0.41 ± 0.33	3.43 ± 1.55	4	(− 1,2)	147.55 ± 318.17	Rostral L	L	29.39 ± 43.07*	70 (14/20)
LTB	−0.51 ± 0.21	4.57 ± 1.05	7	(− 1,3)	119.8 ± 188.58	Rostral L	L	84.94 ± 223.46	70 (14/20)
LFDI	−0.51 ± 0.39	6.39 ± 1.35	19	(− 1,7)	915.73 ± 1255.13	Rostral L	L	1778.85 ± 2201.98	80 (16/20)
LADM	−0.56 ± 0.38	6.53 ± 1.42	19	(− 1,7)	676.94 ± 1693.99	Rostral L	L	1233.58 ± 2197.58	75 (15/20)

Group-level spinal mapping results from Experiment 1 (n = 19) and coil-orientation results from Experiment 2 (n = 20) are presented for each muscle. Values are reported as median ± interquartile range (IQR) across participants. For spinal mapping (experiment 1), the peak site was defined as the stimulation site within the 3 × 10 grid that elicited the highest group-level median sMEP amplitude for each muscle. Peak coordinates represent the corresponding x- and y-coordinates of this peak site. For the coil-orientation analysis (experiment 2), peak orientation was defined as the orientation among the eight tested directions that elicited the highest sMEP amplitude. Peak site was defined as the stimulation location among the left, middle, and right sites that elicited the highest sMEP amplitude. Peak sMEP amplitude represents the median ± IQR amplitude at the optimal orientation/site combination. Peak-orientation frequency represents the proportion of participants whose individual maximal response occurred at the group-level peak orientation. Values in parentheses indicate the number of participants out of the total number tested. For the right FCR muscle, data from 5 of 20 participants were excluded because of background noise contamination; therefore, right FCR results were calculated from 15 participants. L-Left, R-Right. * indicates that the sMEP amplitudes were ≤ 50 µV and were therefore considered non-responses; consequently, the corresponding variables, including peak site, peak direction, and peak response, should be interpreted with caution

### Spinal mapping results

Results indicated spatially distinct excitability profiles across the tested upper limb muscles over 30 stimulation sites (3 columns × 10 rows) mapped over the cervicothoracic spinal region (Friedman test, x-CoG: x_(10)_^2^ = 146.2, *p* < 0.0001; y-CoG: x_(10)_^2^ = 118.8, *p* < 0.0001) (Fig. 4). Post-hoc comparisons revealed further significant differences across mediolateral columns (left vs. middle vs. right) and rostrocaudal rows (row 1–10) of the stimulation grid (all *p* < 0.01). In particular, significant side-to-side differences in mediolateral location were observed for all tested homologous muscles, except the FCR muscle, which was excluded from this analysis (all *p* < 0.0002). Significant differences were also observed between arm and hand muscles (left BB vs. left FDI, *p* < 0.0001; left BB vs. left ADM, *p* < 0.0001), arm and forearm muscles (left FCR vs. left BB, *p* = 0.0006), and forearm and hand muscles (left FCR vs. left FDI, *p* = 0.01; left ECR vs. left FDI, *p* < 0.0001; left ECR vs. left ADM, *p* < 0.0001).

Amplitudes of sMEPs were generally significantly greater at left- than right-column sites (Friedman test, all *p* < 0.001; except for right ADM and right FDI, which showed no sMEPs) and varied systematically along the rostro–caudal axis (Friedman test, all *p* < 0.001; except for right ADM, right ECR, right FDI, right TB, which showed no sMEPs). Overall, arm muscles showed higher responses rostrally (rows 1–3), wrist muscles peaked at mid-rows (rows 3–6), and hand muscles exhibited greater responses caudally (rows 7–10).

### Coil orientation results

Amplitudes of sMEPs were significantly influenced by stimulation sites (left, middle, right) (Friedman test, all *p* < 0.017) and induced current direction (i.e. coil handle orientation angle) (Friedman test, all *p* < 0.001).

**Fig. 5:**
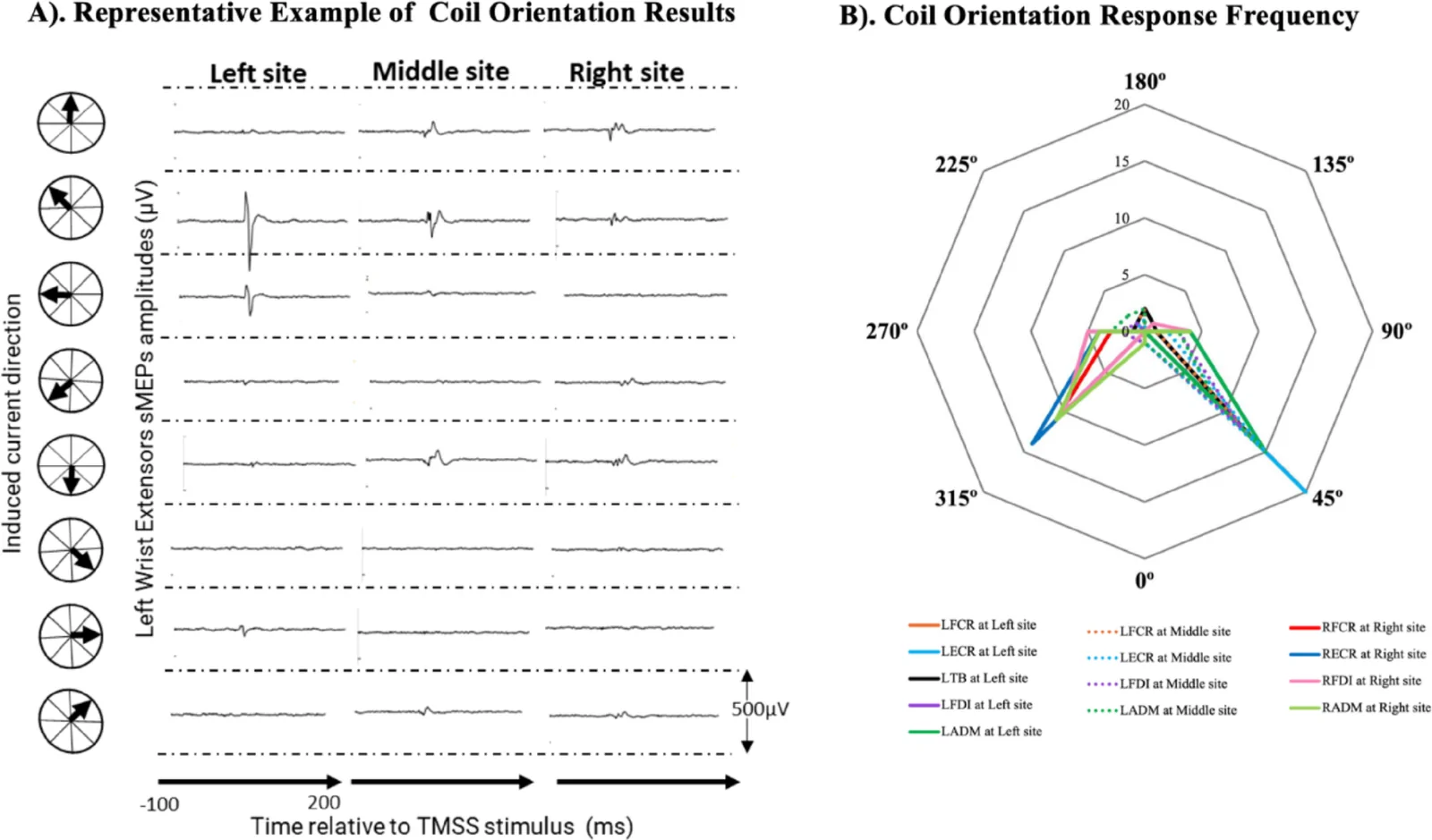
Coil-orientation results. **A** Representative example of coil-orientation responses from one participant with raw sMEP traces, recorded from the left ECR muscle, across the left, middle, and right stimulation sites and eight tested coil orientations. The clock plot on the left illustrates the induced current direction corresponding to each coil orientation. For example, when the coil was oriented at 0°, a rostral current direction was induced (top row). **B** Peak-direction frequency across responsive muscles. The outer labels of each polar plot indicate the tested coil orientation angles, and the values inside the plot indicate the number of participants whose maximal sMEP amplitude occurred at the corresponding orientation. Muscles with no detectable sMEP activity (sMEP amplitudes < 50 µV) were excluded from the graph

Representative left ECR responses from one participant are shown in Fig. 5 for visualization. The left ECR was selected as the representative muscle because the stimulation hotspot and stimulation intensity for the rotation experiment were determined based on activation of this muscle. In this example, the largest sMEP amplitude was elicited when the coil was oriented at 45°, corresponding to a rostral-left induced current direction. At this direction, sMEP amplitudes were greatest at the left stimulation site, intermediate at the middle site, and smallest at the right stimulation site.

**Fig. 6:**
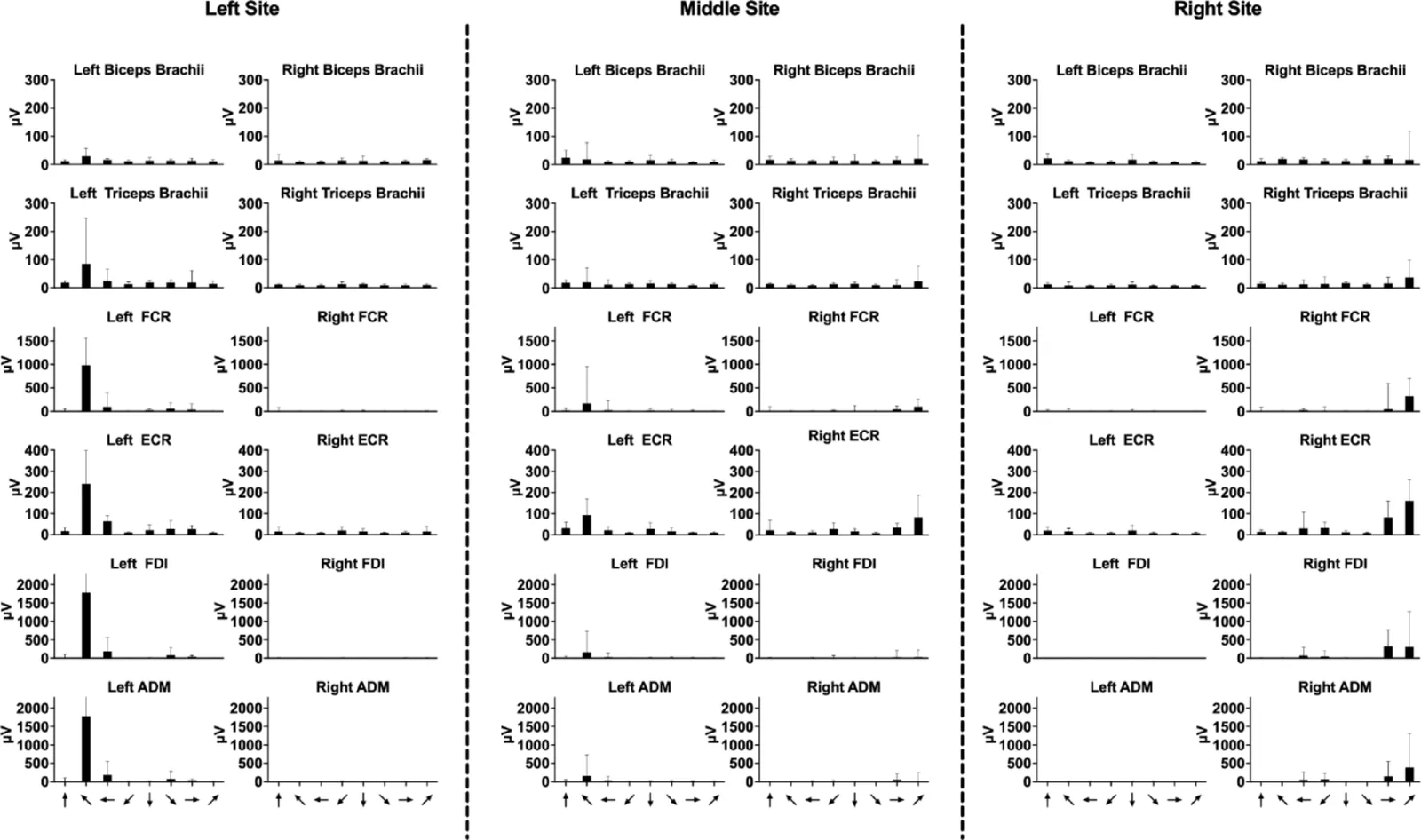
TSMS-induced responses from 12 upper limb muscles elicited by eight coil orientations across three stimulation sites. The arrows underneath the x-axis indicate the TSMS-induced current flow corresponding to each tested coil orientation. Each small panel represents the response of an individual muscle to the 8 examined TSMS-induced current directions, plotted as the median ± IQR across 20 participants. Abbreviations used in the figure include FCR (flexor carpi radialis), ECR (extensor carpi radialis), FDI (first dorsal interosseous), and ADM (abductor digiti minimi)

This site- and orientation-dependent response pattern was consistent with the group-level results shown in Fig. 6, which summarizes TSMS-induced sMEP amplitudes across muscles, coil orientations, and stimulation sites. However, several muscles were non-responsive, defined as sMEP amplitude < 50 µV, and were therefore excluded from further analysis. Specifically, no sMEPs were elicited from the right TB, right ECR, right FDI, or right ADM when stimulated at the left site, or from the left BB, left TB, left ECR, left FDI, or left ADM when stimulated at the right site, across any of the eight tested coil orientations. Among the remaining muscles with detectable responses, left-sided muscles showed significantly larger sMEP amplitudes with the rostral-left induced current direction, corresponding to a 45° coil orientation, whereas right-sided muscles showed significantly larger sMEP amplitudes with the rostral-right induced current direction, corresponding to a 315° coil orientation (all *p* < 0.002). Left-sided muscles consistently showed the largest responses at the left stimulation site, followed by the middle and right sites, whereas right-sided muscles showed the opposite pattern (all *p* < 0.00001).

Overall, responses from left- and right-sided muscles showed a consistent mirror-symmetric pattern (Fig. 5B). For left-sided muscles, the largest proportion of participants showed their maximal sMEP response with the rostral-left induced current direction, corresponding to a 45° coil-handle orientation. Conversely, for right-sided muscles, the largest proportion of participants showed their maximal sMEP response with the rostral-right induced current direction, corresponding to a 315° coil-handle orientation.

## Discussion

Spinal stimulation using electrical methods, either invasively or non-invasively, has shown promising therapeutic efficacy across a range of sensorimotor functions, including independent standing (Rejc, Angeli, Atkinson, et al. 2017), stepping (Harkema et al. 2011; Rejc, Angeli, Bryant, et al. 2017), and walking ability (Angeli et al. 2018); as well as in the restoration of hand function (Powell et al. 2023), hand strength (Freyvert et al. 2018; Inanici et al. 2018), hand sensation (Inanici et al. 2021), and grip force (Zhang et al., 2020). However, concerns have been raised. For example, de Freitas et al. (de Freitas et al. 2021) reported mild-to-moderate pain (3–5/10 on a visual analog scale) during transcutaneous spinal electrical stimulation and limited tolerance with high intensity levels. Magnetic stimulation may help address these limitations by recruiting fewer cutaneous sensory afferents than electrical stimulation, thereby reducing pain or discomfort. Supporting this possibility, Sasada et al. (2021) reported that repetitive transcutaneous magnetic spinal stimulation (rTSMS) was generally well tolerated, with only 10% of participants reporting discomfort at frequencies below 20 Hz over the lumbosacral spine (rTSMS) (Sasada et al. 2021). Thus, as an initial step toward clinical translation, the present study characterized TSMS-evoked spinal responses in healthy individuals and examined how stimulation parameters influenced response distribution across upper-limb muscles.

Given the need for systematic investigation of fundamental stimulation parameters of TSMS, we investigated how coil position and orientation influence motor responses in upper extremity muscles. The present findings demonstrated that coil location and orientation both play significant roles in shaping muscle responses. We observed consistent medial–lateral and rostral-caudal gradients across arm, wrist, and hand muscles. Within the cervicothoracic grid, muscle responses were largest after stimulation of ipsilateral sites, reduced at the midline sites, and smallest at contralateral sites. A rostral-caudal response was also evident, as the arm muscles exhibited a rostral advantage, wrist muscles peaked in the mid rows, and hand muscles produced larger responses with more caudal stimulation. Across the eight tested current directions, a rostral-left orientation elicited the largest responses in left-sided muscles, whereas a rostral-right orientation was most effective for right-sided muscles. This effect was consistent across the three cervicothoracic sites, with maximal responses ipsilaterally, moderate responses at midline, and minimal responses contralaterally, aligning with prior medial–lateral findings. These findings have important implications for establishing standardized techniques and protocols for TSMS in therapeutic and assessment applications.

### Effects of stimulation location on sMEPs

In the spinal mapping results, we found a significant medial–lateral difference that provided convergent evidence regarding the locus of stimulation. Our findings suggest that stimulation at lateral sites positions the induced field closer to the spinal nerve roots, which may recruit motor pools via multiple spinal and supraspinal reflex pathways and yield stronger muscle responses. Responses therefore followed an ipsilateral > midline > contralateral pattern, consistent with the hypothesis that TSMS predominantly targets the dorsal roots rather than central spinal cord structures (Murray & Knikou 2019). However, confirmation will require additional modeling and imaging studies, as well as an analysis of latency. Additionally, significant differences along the rostral–caudal axis paralleled segmental innervation patterns defined by spinal myotomes: arm muscles peaked near C5–C6, wrist muscles near C6–C7, and intrinsic hand/finger muscles near T1. Together, medial–lateral and rostral–caudal differences reveal a level of spatial resolution that can refine TSMS targeting for assessment and therapeutic applications.

Notably, these effects were more pronounced in left-sided muscles, whereas right-sided responses were comparatively limited. Such predominance may be attributed to the use of a rostral-left induced current (coil handle at 45°) during mapping. A more balanced bilateral representation may have been observed if the right column had been stimulated using a rostral-right current direction (coil handle at 315°), which was optimal for right-sided muscles as indicated in the subsequent orientation testing results.

Beyond the observed mapping results, stimulation intensity must also be considered as an important methodological factor influencing the interpretation of the mapping results. In the present study, stimulation intensity was set at 120% of the BB rMT to reduce the possibility of missing responses across bilateral arm, forearm, and hand muscles. However, whether this intensity level influenced spatial specificity remains unknown, as higher intensity may increase current spread and produce broader response distributions (Thickbroom et al. 1998). In a structured review of cortical mapping studies using transcranial magnetic stimulation (TMS), a similar non-invasive magnetic stimulation approach but with a different target location than TSMS (i.e. cortical vs. spinal), Sondergaard et al. reported that both 110% rMT (49/132 papers) and 120% rMT (51/132 papers) were commonly used (Sondergaard et al. 2021). Kallioniemi and Julkunen (2016) further suggested that 110% rMT may provide greater spatial specificity than 120% rMT, as motor responses elicited at 110% rMT were more comparable to those elicited using an upper-threshold intensity, defined as the lowest intensity at which 100% of responses exceeded 50 μV(Kallioniemi & Julkunen 2016). Therefore, future studies designed to optimize segmental spinal mapping may benefit from systematically comparing stimulation intensities to determine the optimal balance between response reliability and spatial specificity.

### Effects of coil orientation on sMEPs

In the coil orientation results, we found that the largest muscle responses were achieved when the induced current was oriented rostrally and at a 45° angle to the spine, as opposed to rostrocaudal or mediolateral orientations (Mills et al. 1993; Ugawa et al. 1989). This observation aligns with previous findings, as summarized in Table 2.

**Table 2:** Summary of previous TSMS coil orientation studies

N (population)	Present study	Ugawa et al. 1989	Mills et al., 1989	Matsumoto et al. 2009	Veldema et al., 2025
	20 (healthy)	10 (healthy)	6 (healthy)	6 (healthy)	27 (healthy)
Targeted muscles	Bilateral biceps, triceps, wrist flexors, wrist extensors, first dorsal interosseous, abductor pollicis brevis	Bilateral biceps, thenar, extensor digitorum brevis	Bilateral deltoid, brachioradialis, extensor digitorum communis, first dorsal interosseous	Right Abductor hallucis	Bilateral Tibialis anterior
Stimulator & coil	MagVenture MagProx 100, Fig. 8 coil	Magstim 200, flat coil	Magstim 200, figure−8 coil	Magstim 200, figure−8 coil	PowerMAG, double-cone coil
Target location	3 medial–lateral locations over cervicothoracic spine, determined with navigation	C2–T4, by palpation	C5–T1, by palpation	S1, by palpation	T8, by palpation
Tested orientation	8 orientations over 0-360º with 45º increments	Clockwise vs counterclockwise	16 orientations over 0-360º with 22.5° increments	12 orientations over 0-360º with 30° increments	3 orientations over 0–180 º with 90º increments
Optimal orientation	45º ipsilateral upward relative to the horizontal	Clockwise for left-sided muscle, counterclockwise for right-sided muscles	Optimal coil current orientation was ~20° ipsilateral-downward relative to horizontal	~60° upward relative to the horizontal produced the largest responses	Lateral orientation was associated with stronger improvement in balance outcomes bilaterally
Visualization (thick arrows represents the optimal induced current flow at corresponding coil orientation angle)					

This table summarizes stimulation parameters used in the present study alongside those reported in prior TSMS studies examining coil orientation. *Thick arrows indicate the optimal direction of induced current flow corresponding to each coil orientation angle. L: left; R: right. "L" and "R" denote the optimal coil orientation for muscles on the respective side

Ugawa et al. (1989), in one of the earliest investigations in this field, reported that induced currents flowing from the periphery toward the spinal cord elicited stronger responses, a finding supported by subsequent work. Mills et al. (1993) further showed that optimal coil orientations for activating wrist and finger muscles were between 22.5° and 45° downward and toward the midline (Mills et al. 1993). Although this appears directionally opposite to our results, it is important to note that our study used the “reverse” mode to invert the current direction; when accounting for this, our findings were consistent with theirs. Similarly, studies on the lower limbs reported that rostrally directed coil orientations between 30 and 60° were most effective for activating thigh and shin muscles (Maccabee et al. 1996; Matsumoto et al. 2009). Overall, these findings highlight rostral induced current directions as the most effective, aligning with the prior assumption that excitation primarily occurs at the intervertebral foramen (Epstein et al. 1991), and a rostrally directed induced current facilitates a more efficient depolarization of the neurons. Variation in the optimal angles for the coil (~ 20° vs. ~60°) may reflect underlying anatomy, since cervical spinal nerves have a more acute angle than those in the lumbar region. While these prior studies provided important insights regarding the optimal coil orientation, our approach advances this work by adding two additional lateral stimulation sites beyond the spinal midline, enabling muscle activation at lower stimulation intensities.

More importantly, the use of a “biphasic” waveform should be considered when interpreting the current-orientation findings. Biphasic pulses can be conceptualized as two sequential phases with opposite current directions(Sommer et al. 2018). Evidence from motor cortex studies suggest that biphasic pulses can activate directionally selective neuronal populations and that the resulting response may be dominated by the second, reverse phase of the pulse (Sommer et al. 2006). To emphasize this interpretation, coil orientation angles were represented as the corresponding direction of the TSMS-induced current in the figures; for example, a 45° coil orientation was described as producing a rostral-left induced current direction. However, these current-direction labels should not be interpreted as isolating activation produced by a single unidirectional current phase. Therefore, the orientation-dependent effects observed in the present study should be interpreted within the context of biphasic stimulation. Future TSMS studies aimed at optimizing current-direction specificity may benefit from systematically comparing different pulse waveforms, such as monophasic and half-sine waveforms.

### Limitations

One limitation of the present study is that the sample included a wide age range but was primarily composed of young adults. Participants aged 21–30 years accounted for 10 of 19 participants in Experiment 1 and 11 of 20 participants in Experiment 2. Because many clinical conditions relevant to TSMS applications are more common in older adults, the generalizability of these findings to patient populations may be limited. Future studies should include age-matched healthy controls and clinical populations to improve the translational relevance of these findings.

A further limitation relates to potential variability in stimulation targeting. Although targeting was neuronavigated, the absence of spinal MRI reference images may have introduced minor variability, as external landmarks cannot fully capture individual differences in cervical neural anatomy. Neck posture is another potential factor as noted by Mills et al. (1993). For each participant, final positioning was adjusted for individual comfort, which may have led to minor differences in neck flexion and the relative positioning of cervical nerves. Another consideration is neck size, which was not accounted for in the current design; the 3×10 grid may be less precise in larger participants, suggesting that future work could benefit from denser coil placement (e.g., 1 cm mediolateral spacing instead of 3 cm). Finally, the coil orientation increments tested here were relatively coarse, limited to 45° steps. Building on previous reports, future studies may gain additional resolution by testing, for example, within the 22.5°–45° range, using finer steps (e.g., 10°) to better characterize orientation-dependent effects.

In addition to these methodological limitations, we acknowledge the limitation that sMEP latency was not analyzed in the present study. Latency analysis may provide complementary information regarding nerve conduction velocity and may allow for speculation about the potential neural substrates involved in TSMS responses. Reliable latency estimation, however, requires accurate trace-level identification of stimulus onset and sMEP onset timing that was not available in this study. Because a wireless EMG system was used, stimulus artifact that is typically readily apparent within MEP traces could not be consistently identified in the present dataset. Wireless transmission delay further complicated the ability to identify latencies accurately. For these reasons, latency outcomes were not reported.

## Conclusion

This study represents the first step toward establishing TSMS as a noninvasive neuromodulation method for improving motor impairments after stroke. Our findings demonstrate that magnetic stimulation can effectively target spinal circuitry, consistent with earlier research conducted using electrical methods (Knikou 2013b), and suggest its potential to modulate spinal excitability (Inanici et al. 2021, 2018). The ability to selectively activate muscle group and sides underscore the therapeutic promise of TSMS for patients with hemiplegia or hemiparesis, especially in patients who have predominantly unilateral deficits after stroke. Beyond its translational relevance, this study also provides practical and educational guidance for future TSMS research, offering insights into optimal coil placement and orientation depending on experimental or clinical objectives. Collectively, these findings mark a significant step towards developing repetitive TSMS as a neuromodulatory priming intervention to help restore motor function following neurological injury.

## Data Availability

Data will be made available upon request.
